# Probing the Specificity of Fluorescent Deoxyribozymes Using Single-Step Selections and Machine Learning

**DOI:** 10.1021/acschembio.5c00969

**Published:** 2026-04-24

**Authors:** Zuzana Král’ová, Lukáš Išler, Martin Volek, Mônica Jandová, Jaroslav Kurfürst, Edward A. Curtis

**Affiliations:** † Institute of Organic Chemistry and Biochemistry of the Czech Academy of Sciences, Prague 166 10, Czech Republic; ‡ Department of Genetics and Microbiology, Faculty of Science, Charles University in Prague, Prague 128 44, Czech Republic; § Department of Informatics and Chemistry, University of Chemistry and Technology, Prague 166 28, Czech Republic

## Abstract

The ability of proteins and nucleic acids to form specific binding sites for ligands is critical for biological function, and methods to modulate biochemical specificity are important for fields such as enzyme engineering and drug design. Here, we systematically investigated the specificities of self-phosphorylating deoxyribozymes that convert the coumarin substrate 4-MUP into a fluorescent product using biochemical assays, single-step selections, and machine learning. Activity assays using a panel of 20 catalytic motifs and 10 substrates that generate different types of signals when they are dephosphorylated revealed that these deoxyribozymes are extremely specific for 4-MUP. To identify mutations that change specificity, we constructed a library based on a self-phosphorylating fluorescent deoxyribozyme called Aurora. A series of single-step selections yielded variants that react with 4-MUP and the structurally similar substrate diFMUP, but not with the more distinct substrates pNPP and ELF. Pairwise analysis of sequences in the 4-MUP and diFMUP data sets revealed four mutations that modulate Aurora specificity. The effects of these mutations were confirmed using biochemical assays and could be predicted using models developed by machine learning. Taken together, our results show how single-step selections can be used to identify mutations that change the specificity of a deoxyribozyme. They also highlight how machine learning can be used to model complex data sets from *in vitro* selection experiments.

## Graphical abstract

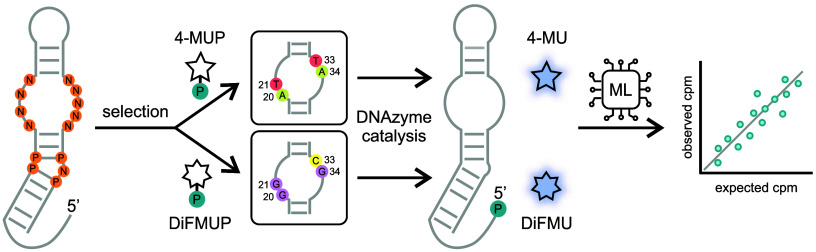

## Introduction

Cells contain many different types of molecules, and mechanisms to distinguish among them are critical for biological function.^1,2^ The ability to tune molecular specificity is also important for fields such as enzyme engineering^3^ and drug design.^4^ From this perspective, developing ways to change the specificity of a biomolecule is an important goal. We are interested in exploring this question in the context of deoxyribozymes (catalytic DNA molecules).^5–9^ These are typically isolated from random sequence libraries using a method called *in vitro* selection,^10–12^ and can have a wide range of biochemical activities and functions. Early studies of deoxyribozyme specificity focused mainly on kinases that phosphorylate functional groups such as the 5′-hydroxyl group of DNA,^13–17^ the 3′-hydroxyl group of DNA,^18^ and the hydroxyl group of tyrosine.^19–21^ ATP or GTP is typically used as the phosphate donor,^13–17,19,20^ but deoxyribozymes that transfer phosphate from PPP-RNA have also been reported.^18,19,21^ Selections in which multiple NTPs or dNTPs are present often yield deoxyribozymes specific for a single substrate (such as GTP), but motifs with broad specificities have also been described.^13,14^ Our lab is interested in developing new deoxyribozymes for diagnostic applications, and recently identified a series of kinase deoxyribozymes that generate chemiluminescent, fluorescent, and colorimetric products.^22–25^ These use monophosphorylated substrates that generate signals in their dephosphorylated, but not phosphorylated forms (Figure 1). In addition to activating the substrate, transfer of the phosphate group to the deoxyribozyme can be used to identify reacted library members during selection and to measure deoxyribozyme rates using a ligation reaction that requires a 5′ phosphate (Figure 1). We have currently discovered three distinct deoxyribozymes using this approach: Supernova, which uses the chemiluminescent substrate CDP-Star,^22,23^ Aurora, which uses the fluorescent substrate 4-MUP,^24^ and Apollon, which uses the colorimetric substrate pNPP.^25^ Additional substrates have been developed that generate signals when they are dephosphorylated (Figure 2a), and identification of deoxyribozymes that use them would provide new possibilities for sensor development.

**Figure 1:**
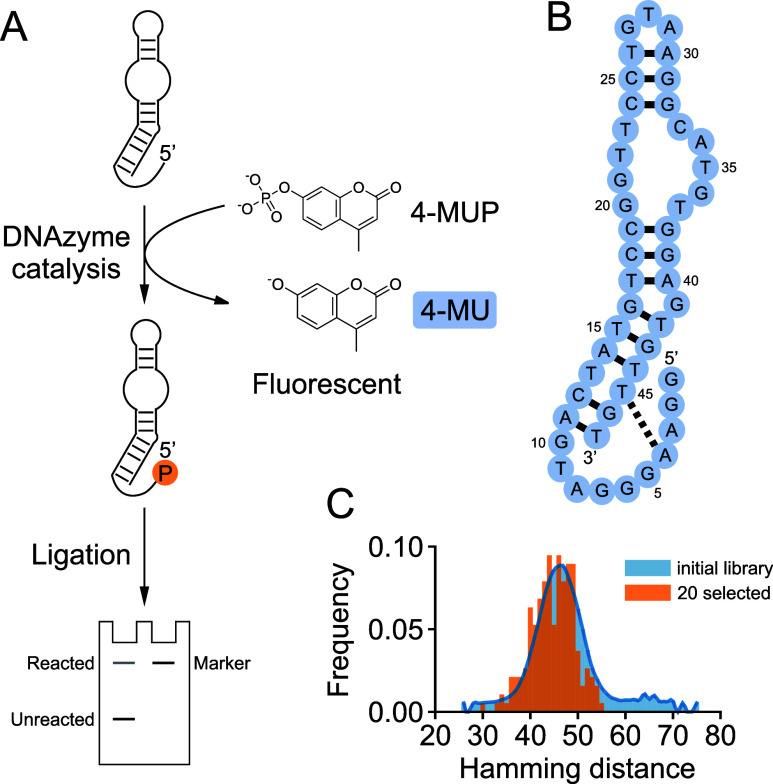
Deoxyribozymes that generate fluorescent signals. (A) Production of fluorescence by deoxyribozymes that transfer the phosphate group from 4-MUP to their own 5′-hydroxyl group. (B) Secondary structure model of the fluorescence-producing deoxyribozyme Aurora.^24^ (C) Sequence diversity of deoxyribozymes isolated in a previous selection for motifs that phosphorylate themselves in the presence of 4-MUP.^24^

**Figure 2:**
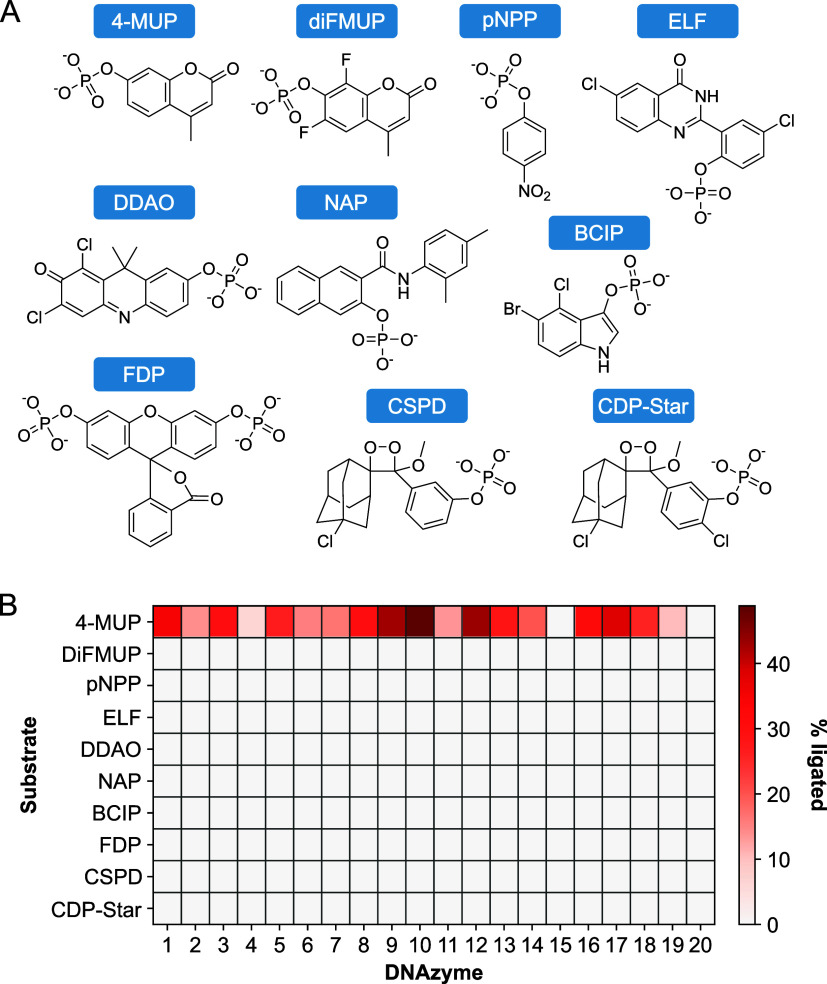
Deoxyribozymes selected to react with 4-MUP are specific for this substrate. (A) Chemical structures of substrates used in these experiments. (B) Ability of 20 deoxyribozymes to react with 4-MUP (the substrate used in the selection in which they were isolated) and nine other substrates. Percent reacted was measured using a ligation assay (which measures the amount of 5′ phosphorylated deoxyribozyme) after a 20-h incubation with substrate.

One goal of the present study was to more systematically investigate the specificities of fluorescent deoxyribozymes previously isolated based on their ability to phosphorylate themselves using 4-MUP (Figure 1).^24^ Activity assays using 20 different catalytic motifs and 10 different substrates (Figure 2a) revealed that these deoxyribozymes are highly specific for 4-MUP. A second objective was to change the specificity of one of these deoxyribozymes, called Aurora, using a series of single-step selections.^26^ These experiments yielded many variants that use the original 4-MUP substrate and some that react with the structurally similar diFMUP, but none that use the more distinct substrates pNPP or ELF. A third aim was to identify mutational changes in Aurora that affect specificity. Four mutations were identified by bioinformatics that change specificity with respect to 4-MUP and diFMUP, and more general patterns were detected using machine learning.^27–31^ Taken together, our results show how single-step selections can be used to both change the specificity of a deoxyribozyme and identify specific mutations that affect specificity. They also indicate that specificity can be modeled using machine learning, and raise the possibility that this approach can be used to explore larger regions of sequence space than are accessible by experimental methods alone.

## Materials and Methods

### Oligonucleotides

Desalted oligonucleotides were purchased from Eurofins Genomics GmbH and purified by 6% urea-PAGE. Bulge libraries 2A-2D were purchased from Integrated DNA Technologies and purified by 6% urea-PAGE. See Table S1 for the sequences of all oligonucleotides used in this study.

### Single-Step Selection of Self-Phosphorylating Deoxyribozymes Using Different Substrates

The library used for single-step selections consisted of four sublibraries designated Aurora 2A-D (Table S1). By equimolar mixing of the four sublibraries, a bulge library containing 6.7 × 10^7^ unique sequences was created. The bulge library was mixed with blocking oligonucleotide (REV1) and Milli-Q water, heated to 65 °C for 2 min and cooled to RT for 5 min. Subsequently, 5× selection buffer was added, followed by one of the substrates (4-MUP, diFMUP, pNPP, or ELF) chosen for single-step selections. Final concentrations in the selection reaction were 1 μM library, 1.5 μM blocking oligonucleotide, 1× selection buffer (50 mM HEPES pH 7.4, 200 mM KCl, 1 mM ZnCl_2_, and 5% DMSO), and 1 mM substrate. The reaction was incubated for 14.4 min in the dark at RT and then stopped by adding 4 M NaCl and 96% ethanol to the final concentrations of 0.3 M NaCl and 70% ethanol. The DNA was then precipitated and, after drying, the pellet was resuspended in 30 μL of Milli-Q water. A short oligonucleotide (oKS065) was ligated to reacted members of the library (which contained a 5′-phosphate). To increase the efficiency of the ligation, a splint oligonucleotide (oKS066) complementary to both oKS065 and the 5′ end of the pool was used. The mixture of all DNA components was heated to 65 °C for 2 min and cooled to RT for 5 min, after which 10× concentrated Standard Ligation Buffer and T4 DNA Ligase (Jena Bioscience) were added. Final concentrations in the ligation reaction were 2 μM DNA library, 2.5 μM oKS065 and splint, 1× Standard Ligation Buffer, and 0.5 Weiss unit of T4 DNA Ligase per 1 μg of DNA. Ligation was performed at 37 °C for 5 min and then stopped by adding 2× bromophenol blue. Ligated molecules were then separated from unreacted ones by 6% denaturing urea-PAGE. The band corresponding to ligated sequences was excised from the gel and the DNA was eluted into 0.3 M NaCl overnight, followed by ethanol precipitation. Ligated product was then purified on a second PAGE gel. Reacted sequences were amplified by PCR using Q5 Hot Start High-Fidelity DNA Polymerase (New England Biolabs). Final concentrations in the PCR were 500× diluted selected pool, 0.5 μM forward and reverse primers, 200 μM dNTPs, 1× Q5 Reaction Buffer, 1× Q5 High GC Enhancer, and 0.02 U/μL of Q5 Hot Start High-Fidelity DNA Polymerase. The resulting double-stranded DNA was purified using a Macherey-Nagel PCR Clean-up kit and then sequenced by Illumina (Novogene).

### Deoxyribozyme Characterization Using the Ligation Assay

To characterize self-phosphorylation reactions, the DNA was first heated to 65 °C for 2 min and then cooled to RT for 5 min. Next, DNA was mixed with 5× concentrated reaction buffer, followed by addition of the substrate. Final concentrations in the self-phosphorylating reaction were 1 μM DNA, 1× buffer (50 mM HEPES pH 7.4, 200 mM KCl, and 1 mM ZnCl_2_), and 1 mM substrate. The reaction was incubated for either 1 or 20 h. It was stopped by adding 0.5 M EDTA to a final concentration of 20 mM. DNA was purified from the reaction components by ethanol precipitation. A short oligonucleotide (oKS065) was then ligated to reacted members of the library, as these sequences contained a 5′-phosphate group. To increase ligation efficiency, a splint oligonucleotide complementary to both the reacted sequence and the short oligonucleotide was included. The mixture of all oligonucleotides was heated to 65 °C for 2 min and allowed to cool to RT for 5 min. Subsequently, 10× concentrated Standard Ligation Buffer and T4 DNA Ligase were added. Final concentrations in the ligation reaction were 2 μM of the DNA oligonucleotide of interest, 2.5 μM short oligonucleotide to be ligated, 2.5 μM splint, 1× Standard Ligation Buffer and 0.5 Weiss unit of T4 DNA Ligase per 1 μg of DNA. Ligation was performed at 37 °C for 5 min and then stopped by adding 2× bromophenol blue. A reaction without substrate served as a negative control. When available, deoxyribozymes that could react with these substrates were used as positive controls. Samples were run on 6% denaturating urea-PAGE gels for 1 h. Gels were stained with GelRed dye and scanned using an Amersham Typhoon imager.

The ligation assay was employed in two parts of this study. First, it was used to evaluate 20 hits obtained from a previous selection performed in our laboratory, in which the Aurora deoxyribozyme was first identified. These hits were tested in the presence of 10 different substrates to assess their substrate specificities (see Table S2 for the names of these substrates). Second, the ligation assay also served as a tool to characterize hits identified in single-step selections described in this paper.

### Sequence Data Processing

Processing of raw reads from all samples consisted of adaptor trimming (cutadapt), read merging (fastq-join), unifying read orientation (bash), 5′ and 3′ primer clipping (cutadapt), pattern matching (bash) and counting of unique sequences (bash). In addition, the 14 variable positions from the 47-nucleotide Aurora deoxyribozyme core sequence were extracted. The variable positions analyzed were: 16, 17, 20, 21, 22, 23, 33, 34, 35, 36, 37, 40, 41, and 42 (numbered according to the convention used in Figure 1). For each data set, sequences were weighted by their read counts to preserve information about sequence abundance in the selected populations. Read counts were normalized to counts per million (CPM) to account for differences in sequencing depth across samples.

### Machine Learning

Data sets from the 4-MUP and diFMUP selections were merged by retaining only the DNA sequences present in both data sets. For each of these 456,835 common sequences, a specificity score was computed as the logarithm of the ratio of the corresponding CPM values, i.e., log­(4-MUP/diFMUP). All sequences were one-hot encoded prior to model training.

The merged data set was randomly split into an 80% training set and a 20% held-out test set. The training set was further downsampled as described in Table S3. For each sampled training subset, an MLPRegressor was trained, with hyperparameters optimized via 3-fold cross-validation using a grid search over the values listed in Table S4. A final model using the selected hyperparameters (“batch_size”: 100; “hidden_layer_sizes”: [200, 200, 200]; “learning_rate”: “constant”; “α”: 0.001; “learning_rate_init”: 0.01, “early_stopping”: True; “max_iter”: 200) was then retrained on each sampled training set and evaluated on the held-out test set.

Model performance was assessed using the Spearman correlation coefficient, the coefficient of determination (*R* ^2^), and mean absolute error (MAE) (Tables S5–S6). In addition to predicting specificity scores, the same modeling procedure was applied to predict 4-MUP CPM values directly, using identical sampling, model and evaluation metrics.

### Data Visualization and Statistical Software

All bioinformatics analyses were performed using Python 3.13. Key packages included: pandas 2.3.1 (data manipulation), numpy 2.2.5 (numerical operations), sklearn 1.6.1 and scipy 1.16.0 (machine learning), and matplotlib 3.8.4 and seaborn 0.13.2 (visualization). Source code for all analysis scripts is available at Figshare with doi: 10.6084/m9.figshare.30801668.

## Results

### Catalytic Motifs Selected for the Ability to Use 4-MUP are Specific for This Substrate

In a previous study we used *in vitro* selection to isolate deoxyribozymes that generate a fluorescent signal by transferring the phosphate group from the substrate 4-MUP to their own 5′-hydroxyl group (Figure 1a,b).^24^ To investigate whether these deoxyribozymes can react with additional substrates, 20 sequences from the selection were chosen for further characterization. These sequences are no more similar to one another than would be expected based on random sampling of the starting library (Figures 1c and S1), suggesting that they represent distinct catalytic motifs. Each of these 20 sequences was incubated with a panel of 10 different substrates (including 4-MUP as a positive control) (Figure 2a). These substrates were chosen because each generates a chemiluminescent, fluorescent, or colorimetric signal when it is dephosphorylated. For each combination of deoxyribozyme and substrate, the extent of self-phosphorylation was assessed after 1 and 20 h using a ligation assay. These deoxyribozymes were extremely specific for 4-MUP, and could not react with either the chemically similar substrate diFMUP or any of the 8 more distinct substrates we tested (Figure 2b). Based on the background of this assay, we estimate that the rates of these deoxyribozymes are in most cases at least 1000-fold faster when using 4-MUP than when using these other substrates. In contrast, the positive control enzyme alkaline phosphatase could react with each of these 10 substrates (Figure S2). These results demonstrate that, despite the lack of a negative selection step in the protocol, deoxyribozymes identified in our previous selection are extremely specific for 4-MUP.

### Single-Step Selection for Aurora Variants That Use New Substrates

We next asked if selection could be used to identify variants of these deoxyribozymes that can use new substrates. A catalytic motif called Aurora was used as a model for these experiments. The secondary structure and 47-nucleotide long minimized catalytic core of Aurora were identified in a previous reselection experiment (Figure 1b).^24^ Another study showed that mutations at position 34 in the asymmetric bulge of Aurora affect its affinity and specificity for 4-MUP,^31^ while unpublished NMR experiments hinted at possible roles of the 16–42 and 17–40 base pairs in substrate binding. To systematically explore the effects of mutations at these and nearby positions on the substrate specificity of Aurora, we generated a library (hereafter referred to as the bulge library) in which each of the 9 positions in the asymmetric bulge and a single bulged G between the 16–42 and 17–40 base pairs were randomized (Figure 3a). In addition, the 16–42 and 17–40 base pairs were encoded by R-Y or Y-R,^31,32^ which facilitates sampling of each of the canonical A-T, T-A, C-G, and G-C pairs as well as G-T and T-G wobbles at these positions (note that C-A and A-C can also occur). This library encoded 6.7 × 10^7^ different sequences, 56.25% of which had the potential to form each of the 11 base pairs in Aurora. Four different single-step selections were performed to search this library for variants that react with either 4-MUP (which served as a positive control), diFMUP (a substrate with a similar structure to 4-MUP), pNPP (a structurally distinct substrate for which deoxyribozymes were previously isolated in our lab^25^), or ELF (a structurally distinct substrate for which deoxyribozymes have not yet been identified). After incubating with substrate, reacted (5′ phosphorylated) library members were tagged by ligation, purified by PAGE, cut from the gel, amplified by PCR, and characterized by high-throughput sequencing (Figure 3b). Sequences were ranked by CPM values (this is the read number of a sequence multiplied by one million and divided by the total number of reads), and selections were evaluated by comparing the distribution of CPM values in the starting library with the distribution after a single round of selection.^26^ In the case of 4-MUP, thousands of enriched sequences were identified, indicating that the library contained many variants that can phosphorylate themselves using this substrate (Figure 3c). Enriched sequences were also detected in the diFMUP selection, but were significantly less abundant than in the 4-MUP selection (Figure 3d). In contrast, no enriched sequences were identified in selections for Aurora variants that react with pNPP (Figure 3e) or ELF (Figure 3f). The most abundant sequence from each selection was also tested for its ability to react with its cognate substrate. Consistent with the results of the selections, the top sequences from the 4-MUP and diFMUP selections were catalytically active, while those from the pNPP and ELF selections were not (Figure 3c–f). These results indicated that mutations at the 14 positions sampled in the bulge library can change the substrate specificity of Aurora, but only to a limited extent.

**Figure 3:**
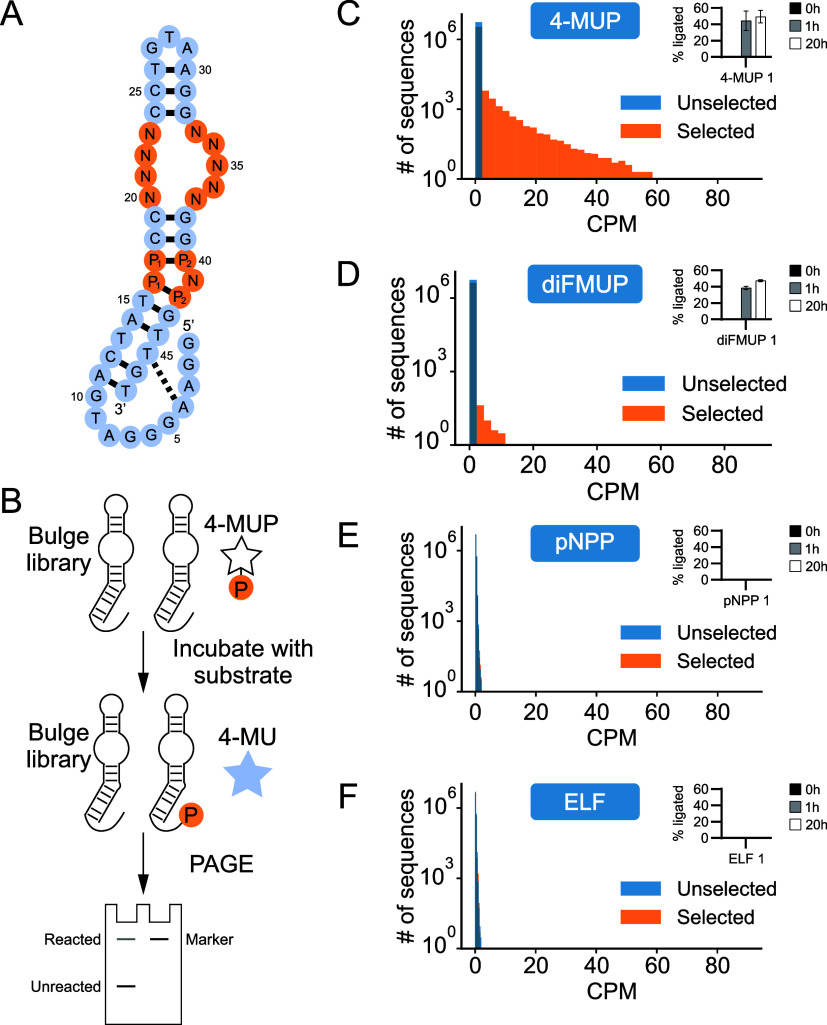
Single-step selection for Aurora variants that can use new substrates. (A) Bulge library based on Aurora. P_1_–P_2_ = R-Y or Y-R, R = A or G, Y = C or T, and N = A, C, G, or T. This library was encoded by 4 different oligonucleotides, each of which contained a different combination of R-Y and Y-R at the 16–42 and 17–40 base pairs.^31,32^ (B) Single-step selection protocol used to identify self-phosphorylating deoxyribozymes. (C) Result of single-step selection for Aurora variants that react with 4-MUP, with the activity of the most abundant sequence in the selected library shown in the inset. (D) Same as panel C, but using the substrate diFMUP. (E) Same as panel C, but using the substrate pNPP. (F) Same as panel C, but using the substrate ELF.

### Identification of Mutations That Affect Substrate Specificity

Our next goal was to evaluate the effects of different mutations on the specificity of Aurora with respect to 4-MUP and diFMUP. To do this, we first made a graph showing the CPM value of each sequence in the library after selection with 4-MUP on the *x*-axis, and its CPM value after selection with diFMUP on the *y*-axis (Figure 4a). This indicated that, while many sequences in the library had higher CPM values after selection with 4-MUP than after selection with diFMUP (these correspond to points to the right of the dotted line in the graph), few had higher CPM values after selection with diFMUP than after selection with 4-MUP (these correspond to points to the left of the dotted line in the graph). Furthermore, among sequences with high CPM values (for example, ≥ 10) none preferred diFMUP over 4-MUP (Figure 4a). To identify mutations most strongly correlated with these changes in specificity, we made a series of versions of Figure 4a in which a single position was highlighted, and each of the possible mutations at this position was shown in a different color. Examination of these graphs showed that mutations at positions 33 were most strongly correlated with the changes in specificity observed among library members, while position 34 (identified in a previous study from our group^31^) was also important (Figures 4b and S3–S4). Further analysis revealed that positions 20 and 21 are important in rare sequence backgrounds (Figure S4). To test the effects of these mutations, each was characterized in a background in which specificity was expected to change significantly based on analysis of CPM values. One of these variants (20G 21G 33T 34T) corresponds to the most abundant sequence from the 4-MUP selection, while another (20G 21G 33C 34T) corresponds to the most abundant variant from the diFMUP selection. These experiments confirmed our bioinformatic analyses, and showed that specificity (defined here as the amount of ligated product after reaction with 4-MUP divided by the amount after reaction with diFMUP) ranged from 1.4-fold (a slight preference for 4-MUP) to 313-fold (a strong preference for 4-MUP) among the sequences we tested (Figure 4c). In addition, point mutations at each of these four positions significantly changed the specificity of Aurora: the 33T to C mutation changed specificity by a factor of 8.9, the 20A to G mutation changed specificity by a factor of 223, the 21T to G mutation changed specificity by a factor of 114, and the 34C to G mutation changed specificity by a factor of 32 (Figure 4c). These results indicate that even positions which are far from one another in the primary sequence and secondary structure can modulate the substrate specificity of Aurora (Figure 4d). The simplest interpretation is that these positions form part of the substrate binding pocket in the three-dimensional structure of the deoxyribozyme, but structural studies will be needed to confirm this hypothesis.

**Figure 4:**
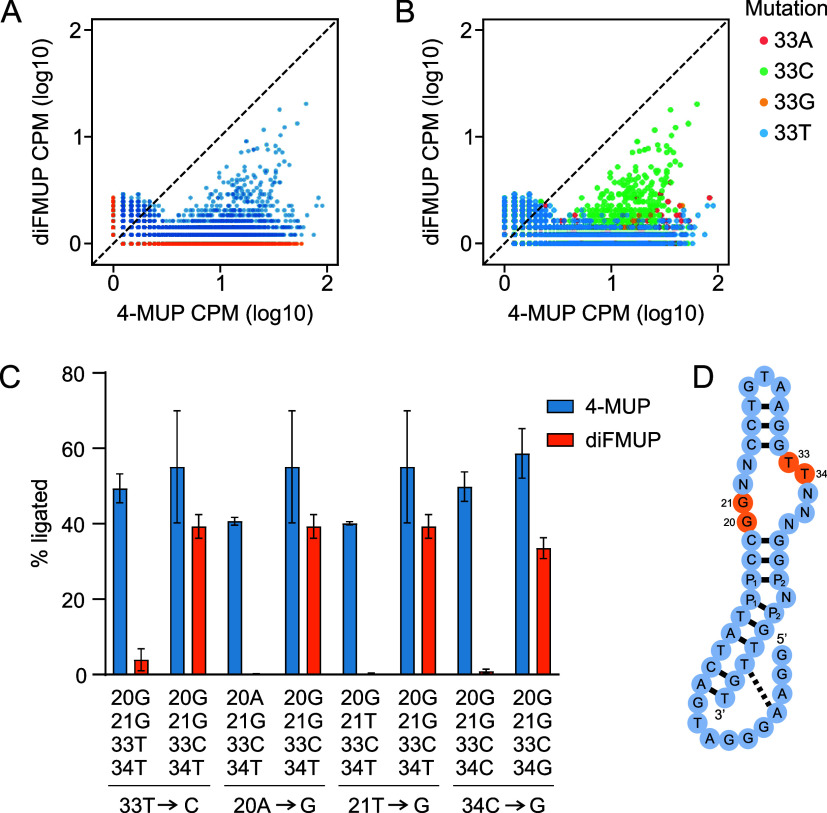
Identification of mutations that allow Aurora to distinguish the similar substrates 4-MUP and diFMUP. (A) Graph showing CPM in the 4-MUP selection on the *x*-axis and CPM in the diFMUP selection on the *y*-axis. Sequences that only occur in one of the two data sets are shown in orange. (B) Identification of mutations that affect substrate specificity. This example shows the effects of mutations at position 33 on specificity with respect to 4-MUP and diFMUP. (C) Effects of mutations at positions 33, 20, 21, and 34 on the ability of Aurora to use 4-MUP and diFMUP. Activity was measured using a ligation assay. (D) Positions important for distinguishing 4-MUP and diFMUP mapped onto the secondary structure of Aurora. See also ref 31.

### Modeling Biochemical Activity and Specificity by Machine Learning

Recent studies indicate that machine learning can be used to model sequence fitness landscapes with data from *in vitro* selection experiments.^27–31^ Such approaches have enormous potential because the number of possible variants of many functional motifs is too large to fully characterize using experimental methods alone. However, the relationship between the type of library and the quality of the model has not been systematically explored. We recently showed that selection data sets from an Aurora secondary structure library^32^ can be used to train models that make it possible to quantitatively predict CPM values of library members using only ∼3% of the data set (and 0.795% of the encoded sequence space) as a training set.^31^ Here we asked whether this approach can be extended to a different type of library (which contained 10 fully randomized positions, two mutagenized base pairs, and was expected to contain a significantly smaller fraction of sequences consistent with the sequence requirements of Aurora than the secondary structure library). We investigated this with respect to two different outputs (Figure 5a). In one case, we asked if models could be developed that predict CPM values of sequences from the 4-MUP data set. In the second case, we asked whether models could be developed that predict the specificities of sequences with respect to the substrates 4-MUP and diFMUP. To address both of these questions, we constructed training sets of various sizes by randomly sampling sequences common to the 4-MUP and diFMUP selections. Models trained on these sampled subsets were then evaluated by predicting CPM values and sequence specificities in held-out test sets. Although the results indicated that reliable predictions could be obtained for both CPM values (*R* ^2^ > 0.5; Figure S5 and Table S5) and to a lesser extent for specificities (*R* ^2^ > 0.2; Figure S6 and Table S6), the model used to predict specificity generated predictions with an apparent lower bound. This is likely related to two imbalances in the training data. First, the 4-MUP data set contains many more enriched sequences than the diFMUP data set. Second, both data sets contain many sequences with low read numbers and few with high read numbers. These biases skew the log_2_(CPM ratio) distribution heavily toward positive values, which is reflected in the model rarely predicting strong diFMUP specificity. Developing these models required approximately 12.5% of the training set, corresponding to approximately 0.068% of the encoded sequence space. Below this threshold, predictive power significantly decreased. Predictions of specificity scores were substantially less accurate than predictions of CPM values, which can be best appreciated by comparing the maximum (no sampling) *R* ^2^ obtained for CPM predictions (*R* ^2^ = 0.85; Figure 5b) with that obtained for specificity predictions (*R* ^2^ = 0.28; Figure 5c). For consistency and direct comparability, all analyses used the same subset of sequences limited to those shared between 4-MUP and diFMUP data sets. Although we do not know what patterns are being recognized by these models, three observations suggest that they are complex. First, pairwise comparisons of the best predicted sequences show that virtually all contain mutations in unpaired regions (Tables S7–S8), indicating that these are not simply variants of Aurora in which base pairs have changed. Second, large numbers of sequences (∼12.5% of the training set) are needed to construct good models (Figures S5–S6 and Table S3). Third, simpler models in which CPM values are predicted based on nucleotides frequencies at individual positions or base pairs perform significantly worse than models developed by machine learning (Figure S7). Taken together, these results indicate that models trained using single-step selection data sets from bulge libraries of the sort used here can yield similar results to those trained using data sets from secondary structure libraries. They also show that specificities of library members can be modeled to a limited extent from such data.

**Figure 5:**
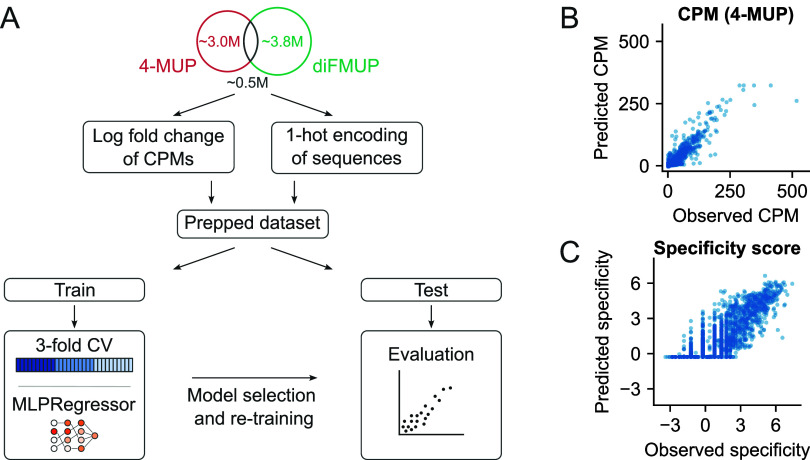
Predicting activities and specificities by machine learning. (A) Machine learning workflow. (B) Predicted and experimentally observed CPM values calculated from the 4-MUP data set (*R* ^2^ = 0.85). (C) Predicted and experimentally observed specificity scores calculated from the 4-MUP and diFMUP data sets (*R* ^2^ = 0.28).

## Discussion

Proteins and nucleic acids can contain binding sites for ligands that determine the specificities of biochemical pathways, and methods to tune the selectivities of these binding pockets are important for fields such as enzyme engineering and drug design.^3,4^ One goal of the current study was to investigate the specificities of deoxyribozymes previously identified based on their ability to react with the substrate 4-MUP.^24^ These deoxyribozymes turned out to be highly specific for 4-MUP, and could not utilize either the structurally similar substrate diFMUP or 8 more structurally distinct substrates. Our results are consistent with previous studies in the group, which have shown that deoxyribozymes selected to use CDP-Star^22^ or pNPP^25^ are also highly specific for these substrates. They are also consistent with results from other groups, which have demonstrated that functional nucleic acid motifs with highly specific binding sites can sometimes be identified even in the absence of specific selective pressures. Perhaps the most remarkable example is an RNA aptamer that binds theophylline 10,000-fold better than caffeine.^33^ This level of discrimination is impressive given that these two ligands differ by a single methyl group. Additional examples were identified in a study in which the specificities of 11 structurally distinct GTP aptamers were characterized using a panel of 16 different GTP analogs.^34^ The affinities of these 11 aptamers were often more than 100-fold higher for GTP than for the GTP analogs tested, and this included aptamers that can distinguish pairs of ligands (such as guanosine and 1-methyl-guanosine) that differed by a single methyl group. A tiny G-quadruplex GTP aptamer isolated from a genome-derived nucleic acid library can also readily distinguish GTP from analogs like 7-methyl-GTP,^35^ as can some self-phosphorylating kinase deoxyribozymes.^17^ These and many other examples demonstrate that negative selection is not always needed to obtain functional nucleic acids with selective binding sites. However, this depends on both the desired specificity of the motif and the most common mechanism of binding in the library. In the case of GTP aptamers, for example, most motifs appear to recognize the Watson–Crick face of guanine. For this reason, negative selection was not needed to obtain aptamers that can distinguish guanosine from analogs like 1-methyl-guanosine, but would be required to identify aptamers that can effectively distinguish GTP from GMP or GTPγS.^34^ In the case of the fluorescent deoxyribozymes analyzed here, the most common mode of binding likely involves contacts with a moiety that occurs in both 4-MUP and diFMUP, but not in the other substrates tested. In contrast, the protein enzyme alkaline phosphatase uses a distinct recognition strategy which enables it to react efficiently with each of the 10 substrates we investigated.

A second goal of this study was to change the substrate specificities of deoxyribozymes that use 4-MUP. This was investigated in the context of Aurora, a deoxyribozyme for which the secondary structure and minimized catalytic core is known.^24^ A library was constructed in which positions known or thought to be involved in substrate binding were mutated, and a series of single-step selections were performed to search for variants that could react with several new substrates. Analysis of these data sets revealed one known mutation and three new mutations that change the specificity of Aurora. These four positions occur on opposite sides of a bulge in the deoxyribozyme, and we hypothesize that they form part of a binding pocket for the substrate. While mutations at these positions make it possible for Aurora to use diFMUP more efficiently than previously characterized constructs, no variants were identified that could use the less similar substrates pNPP or ELF. This suggests that the evolvability of the substrate binding pocket of Aurora is limited (although it is important to note that only 14 of the 47 positions in Aurora were mutated in this library). Previous attempts in our group to identify variants of the Supernova deoxyribozyme that could use new substrates mostly yielded deoxyribozymes with new folds rather than variants of Supernova with altered specificities.^24,25^ Similar results have been obtained with ribozymes,^36^ and sometimes (but not always) with aptamers.^37–41^ These results indicate that in some cases it can be easier to find a completely new catalytic fold or substrate binding pocket in DNA or RNA sequence space than to modify an existing one for a new function.

A final goal was to use machine learning to model the relationship between Aurora sequence and function as well as between Aurora sequence and specificity. Such approaches have great potential because they can possibly be used to explore larger regions of sequence space than are accessible using experimental methods alone. For this reason, we and others have attempted to develop models that can predict the properties of new sequences.^27–31^ However, many fundamental questions remain. These include the best method of library design, the size of the training set relative to encoded sequence space needed to achieve accurate modeling, and the extent to which a single workflow can be applied to functional motifs with different activities and folds. In a previous study we showed that, when using a secondary structure library^32^ encoding approximately 10^6^ different sequences consistent with the constraints of Aurora, training sets made up of approximately ∼3% of these sequences (0.795% of the encoded sequence space) were sufficient to generate models that can predict the activities of the remaining library members.^31^ To learn more about how to develop such models, here we generated our training set using a different type of library. This contained 10 fully randomized positions (as well as two base pairs encoded by R-Y or Y-R), and therefore was expected to contain a significantly lower fraction of active sequences than our previously described Aurora secondary structure library.^31^ Despite this, reasonably good predictions could still be attained using a minimum of ∼12.5% of the data for training (corresponding to 0.068% of the total sequence space). Models trained using our data set could also be used to predict specificity scores with respect to the substrates 4-MUP and diFMUP, although less reliably. The difference between predictive power with respect to CPM values and specificities is likely due in part to the fact that the diFMUP data set contains a significantly lower number of enriched sequences compared to the 4-MUP data set. It could also reflect underlying functional constraints, such as the possibility that patterns that determine specificity are more complex than those which determine activity. These results show how selection and machine learning can be combined to model deoxyribozyme activity and specificity. This approach has the potential to increase the amount of sequence space sampled in selection experiments, and we suggest that it represents a promising future direction in the field.

## Supplementary Material



## Notes

Sequences of all deoxyribozymes used in this study, as well as additional experimental details (PDF)
