# Supplementary material for: Probing the Specificity of Fluorescent Deoxyribozymes Using Single-Step Selections and Machine Learning
Source: ACS Chem Biol. 2026 Apr 24;21(5):951–9. doi: 10.1021/acschembio.5c00969 (PMC13184989; doi:10.1021/acschembio.5c00969)
Supplement: Supplementary file 1 [file cb5c00969_si_001.pdf]

Probing the specificity of fluorescent deoxyribozymes using single-step selections and machine learning

Zuzana Kráľová†, Lukáš Išler†, Martin Volek, Mônica Jandová, Jaroslav Kurfürst, and Edward A. Curtis*

Institute of Organic Chemistry and Biochemistry of the Czech Academy of Sciences
Flemingovo nám. 2.
166 10 Praha 6
Czech Republic

Phone: +420 733 169 654
E-mail: curtis@uochb.cas.cz

*Corresponding author
†Co-first authors

Keywords: Deoxyribozyme, in vitro selection, Aurora, fluorescence, machine learning

Table S1. Sequences of all oligonucleotides used in this study.

library Aurora 2A	GGAAGGGATGACTATYYCCNNNNCCTGTAAGGNNNNNGGRNRGTTGTCTCATGAACTATCCGCTGGA
library Aurora 2B	GGAAGGGATGACTATRRCCNNNNCCTGTAAGGNNNNNGGYNYGTTGTCTCATGAACTATCCGCTGGA
library Aurora 2C	GGAAGGGATGACTATRYCCNNNNCCTGTAAGGNNNNNGGRNYGTTGTCTCATGAACTATCCGCTGGA
library Aurora 2D	GGAAGGGATGACTATYRCCNNNNCCTGTAAGGNNNNNGGYNRGTTGTCTCATGAACTATCCGCTGGA
REV1	TCCAGCGGATAGTTCATGAG
oKS065	ACCGCTCAGGTGTAGTATCA
oKS066	GTCGCCATCTCTTCCTGATACTACACCTGAGCGGT
85_Hit_1	GGAAGAGATGGCTACTACCAAGCTACGTGGCCTAATATCATCATGGCCCCGGACCTCAAGAAGTAAGACGTTGGAATGGCGCATG
85_Hit_2	GGAGGAGATGAAGACGTCCGCGGCCCAAGCTCGTCTATGCTCAATGCGATCTGCTCCTAGGTCGATTTAGTTGCGACGCGTGCGC
85_Hit_3	GGAAGAGAGCGTGCCGTCAGACAGATGAAACTGAAACACCTCAGTGTGAAGTGCTGCGGTGGGCTGTGACCCCCTATGACGGCTC
85_Hit_4	GGAAGAGATGTAGACAAGGCAGGCCCCATTCCTTGCATACTTAGTGCCTAGGACCCCGTGGGGCAGTAACTAGGGATGGAGTTGC
85_Hit_5	GGTAGAGAGGACGACGGCCCCGGAAGATTTCTGAATATCCTCCGTGCGCTTTATCGCAGGGGGGATTTAGTTTCGATGGAGCGTC
85_Hit_6	GGAAGATAATGCGAGGAGGCAGGTCCCATACCAGAGCGTCTAAGGTCGCACTACCGAATGGTCGTGAGACTCTGGATGGAGTTGC
85_Hit_7	GGAAGAGACAGGGAGGAGATAGGCCCCATGCAGTACCTCCCAGATGCGACGATCCACAGGGTGGTACGATATCGCATGGAGCTAT
85_Hit_8	GGAAGAGCCGGCGATGAAATAGAGACGAGTTATAATGGTCCAATTCCGCAGGTCCGCCGGGCGGTCTGACTGAGGAGAGCGCATT
85_Hit_9	GGAAAAGAGCGCGACAATATTCGTACGATGCATGTACACGCCAGTCCGTAGACCCAAGTGGGGGAGTGTCTTGGGCTGGGGGCAA
85_Hit_10	GGAAGAGATGACCAGGGCAGCGGGACGCTGACGAATTTTCTCACTATGTCCGGGACCCGAGGGGCGTGAGGAGTGTTGTGCAATT

85_Hit_11	GGGAGAGATGGCGATTAGAGACTTACGTTGAGGG ATAGCCTCATTGATTATGGCCCCAGTGGAATGTAA AATAGACTGGAGCATA
85_Hit_12	GGAAGAGTAGGCAATGTCCCACGTACGGTACCGC ATATAGTGGGTCCGGACTGCTGAAAGGAGAGGAG CGTTGCGAAAGTGTGTT
85_Hit_13	GGAAGAGATGGAGAAGACTTGGGAAGCATTCCCA AAATTCCCAGTTATCATGGTCCCAGACGCACTTGA CTAGCTATGGTGCATG
85_Hit_14	GGAAGAGATCAGGACCATCATGGCCCCATACCGA AGGACCACAATGACCATGTCAGATATATGTAAAT ACTTGGTATGGAGCATG
85_Hit_15	GGAAGAGAGGGAGATGACGCAGGTTCCATGGGG AATACCCTCAGAGCGCAGGAACGAAGTTGCGAGT GACGTCCGATGGCGCGCC
85_Hit_16	GGAAGAGTTGTCGACGTCCAAGAGACGAGGTCTA TTCGCCTACAACCCGAGCTTAGCGCACGGAAGTG ACTTGAAATGGGTACCC
85_Hit_17	GGAGTAGATGACGACCACACATTAGGGGTGTAGA GTGTACTCACGCCCCAGTGCCGGAGCGGCGAGCG ACGTGGTATGCGGAGTA
85_Hit_18	GGAAGAGATGGCGCAGTCACCATAACGGTCCCGA ATGTCCACACCGCGCAGGGCAGCAGGTGCGAATG GATTGAGATTGTGCGTT
85_Hit_19	GGAAGGGATGTCGAGGACACCAGTACGGTCCCGA ACATCGTCAGAGCGCATGGCCGTACGTGGAAGTG AAGTTGTTCGGAGCGTA
85_Hit_20	GGAAGGGATGGCTGTCCCCAAGGGATGATGCTAA TTATCTTCAGACGGCCGGTACTCCGGCGGGATTGA TTTGGGCTGGGGAATC
Aurora2	GGAAGGGATGACTATGTCCGGTTCCTGTAAGGCA TGTGGAGTGTTGT
4-MUP 1 (Aurora20G21G33T34T)	GGAAGGGATGACTATGTCCGGTTCCTGTAAGGTTT GTGGAGTGTTGT
diFMUP 1 (Aurora20G21G33C34T)	GGAAGGGATGACTATGTCCGGTTCCTGTAAGGCT TGTGGAGTGTTGT
pNPP 1	GGAAGGGATGACTATATCCTTTCCTGTAAGGTTT ATGGATTGTTGT
ELF 1	GGAAGGGATGACTATGTCCATTTCCTGTAAGGTTT AAGGATTGTTGT
Aurora 20A33C34T	GGAAGGGATGACTATGTCCAGTTCCTGTAAGGCT TGTGGAGTGTTGT
Aurora 21T33C34T	GGAAGGGATGACTATGTCCGTTTCCTGTAAGGCTT GTGGAGTGTTGT
Aurora 20G21G33C34G	GGAAGGGATGACTATGTCCGGTTCCTGTAAGGCG GGTGGAGTGTTGT

Aurora 20G21G33C34C	GGAAGGGATGACTATGTCCGGTTCCTGTAAGGCC GGTGGAGTGTTGT

Table S2. List of phosphorylated substrates used in this study.

substrate	abbreviation	type of signal
4-methylumbelliferyl phosphate	4-MUP	fluorescent
6,8-difluoro-4-methylumbelliferyl phosphate	diFMUP	fluorescent
2-(5'-chloro-2-phosphoryloxyphenyl)-6-chloro-4(3H)-quinazolinone	ELF	fluorescent
9H-(1,3-dichloro-9,9-dimethylacridin-2-one-7-yl) phosphate	DDAO	fluorescent
fluorescein diphosphate	FDP	fluorescent
3-(2,4-dimethylphenylcarbamoyl)naphthalen-2-yl phosphate	NAP	fluorescent
p-nitrophenyl phosphate	pNPP	chromogenic
5-bromo-4-chloro-3'-indolylphosphate p-toluidine	BCIP	chromogenic
2-chloro-5-(4-methoxyspiro{1,2-dioxetane-3,2′-(5′-chloro)tricyclo[3.3.1.13,7]decan}-4-yl)-1-phenyl phosphate	CDP-Star	chemiluminescent
3-(4-methoxyspiro{1,2-dioxetane-3,2'-(5'-chloro)tricyclo[3.3.1.13,7]decan}-4-yl)phenyl phosphate	CSPD	chemiluminescent

Table S3. Sampling sizes used for training sets in machine learning.

	Sampling name	Sampling fraction	Seqspace sampling	Sequences
0	sample_1	1.000000	0.005446	365468
1	sample_2	0.500000	0.002723	182734
2	sample_3	0.250000	0.001361	91367
3	sample_4	0.125000	0.000681	45683
4	sample_5	0.062500	0.000340	22841
5	sample_6	0.031250	0.000170	11420
6	sample_7	0.015625	0.000085	5710
7	sample_8	0.007813	0.000043	2855
8	sample_9	0.003906	0.000021	1427
9	sample_10	0.001953	0.000011	713

Table S4. Hyperparameter values used for machine learning.

Hyperparameter	Values Tested
batch_size	200; 100
hidden_layer_sizes	[50, 50]; [50, 50, 50]; [200, 200]; [200, 200, 200]; [400, 400]; [400, 400, 400]
alpha	0.0001; 0.001
learning_rate_init	1×10^{-4}; 1×10^{-3}; 1×10^{-2}
learning_rate	constant
early stopping	TRUE
max_iter	200

Table S5. CPM modeling results.

	sampling_name	spearman	R^2	MAE
0	sample_1	0.163	0.857	0.702
1	sample_2	0.239	0.882	0.573
2	sample_3	0.250	0.834	0.566
3	sample_4	0.230	0.737	0.575
4	sample_5	0.193	0.539	0.652
5	sample_6	0.070	0.615	0.737
6	sample_7	0.147	0.276	1.057
7	sample_8	0.090	0.087	0.777
8	sample_9	0.086	0.215	0.998
9	sample_10	0.034	0.138	0.842

Table S6. Specificity modeling results.

	sampling_name	spearman	R^2	MAE
0	sample_1	0.199	0.285	0.293
1	sample_2	0.195	0.285	0.307
2	sample_3	0.102	0.280	0.279
3	sample_4	0.108	0.267	0.297
4	sample_5	0.084	0.242	0.298
5	sample_6	0.080	0.204	0.284
6	sample_7	0.072	0.126	0.314
7	sample_8	0.067	0.086	0.327
8	sample_9	0.078	0.099	0.341
9	sample_10	0.063	0.045	0.327

Table S7. The most active sequences predicted by machine learning in the 4-MUP dataset contain mutations in both base pairs and unpaired regions of the secondary structure. Sequences with the top 100 predicted CPM values were analyzed. All possible pairs of sequences were generated, and the number of mutations in base pairs and unpaired regions was determined.

	**Mutations in base pairs**		
**Mutations in unpaired regions**	**0**	**1**	**2**
**0**	0	4	0
**1**	28	21	3
**2**	121	102	9
**3**	430	369	22
**4**	763	595	30
**5**	817	549	35
**6**	433	256	39
**7**	156	92	25
**8**	25	19	3
**9**	3	1	0
**10**	0	0	0

Table S8. The most active sequences predicted by machine learning in the 4-MUP dataset contain mutations in both base pairs and unpaired regions of the secondary structure. Same as Table S7, but for sequences with the top 1000 predicted CPM values.

	Mutations in base pairs		
**Mutations in unpaired regions**	**0**	**1**	**2**
**0**	0	60	16
**1**	357	596	271
**2**	2,559	3,919	1,515
**3**	9,347	14,214	5,006
**4**	22,212	31,385	11,580
**5**	34,510	47,086	19,197
**6**	35,859	50,621	26,323
**7**	24,680	40,942	29,070
**8**	10,342	23,702	24,197
**9**	2,083	9,329	12,820
**10**	264	2,201	3,237

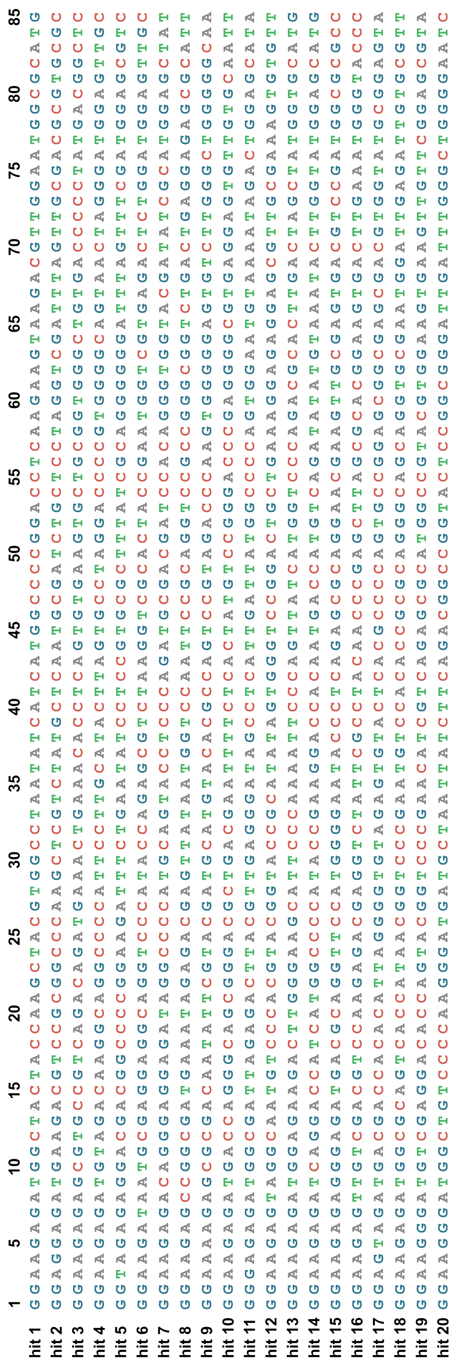

Figure S1. **Sequence alignment of the 20 deoxyribozymes from the 4-MUP selection used in these experiments.**

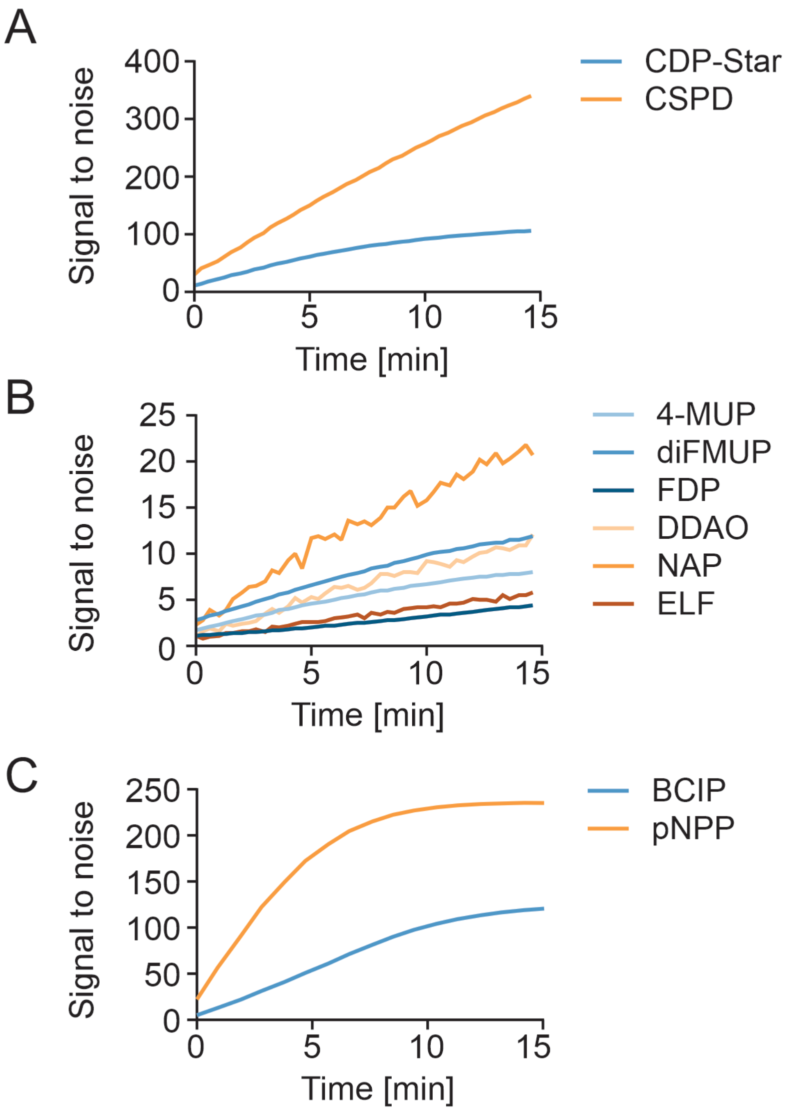

Figure S2. Control experiment showing the reaction of each of the 10 substrates used in these experiments with the protein enzyme alkaline phosphatase. A) Reaction with the chemiluminescent substrates CDP-Star and CSPD. B) Reaction with the fluorescent substrates 4-MUP, diFMUP, FDP, DDAO, NAP, and ELF. C) Reaction with the colorimetric substrates BCIP and pNPP. Reactions contained 10 μM substrate, 1× Fast AP Buffer and either 2×10^{-5} U/μl of alkaline phosphatase (panel A, B) or 2×10^{-4} U/μl of alkaline phosphatase (panel C).

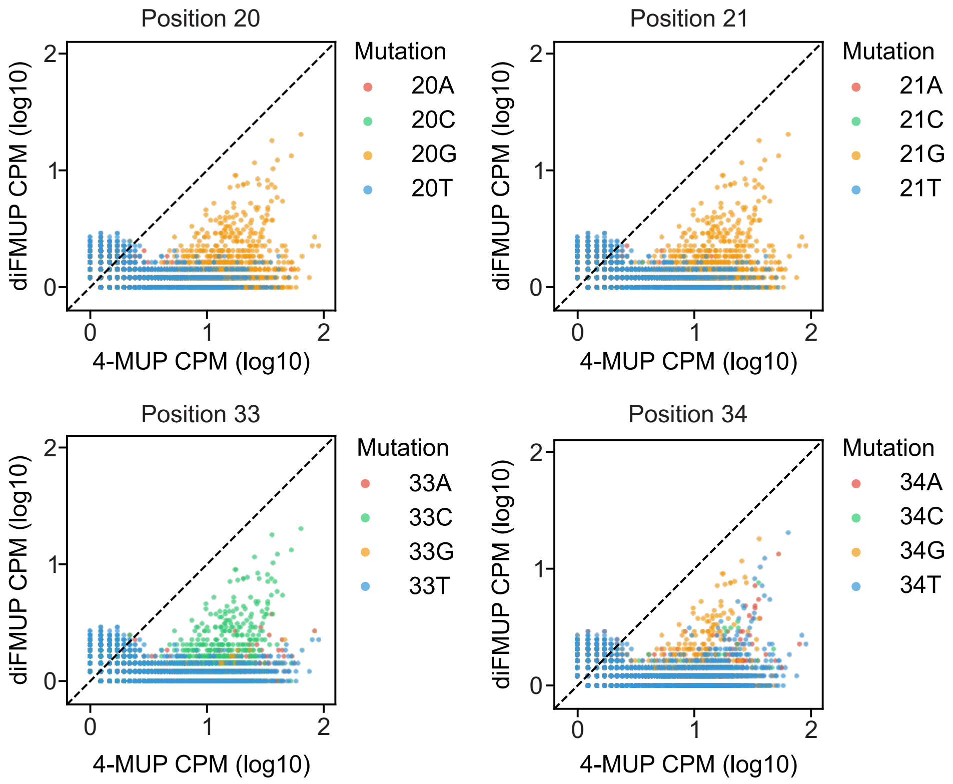

Figure S3. Correlation of mutations at different positions with changes in Aurora specificity with respect to 4-MUP and diFMUP.

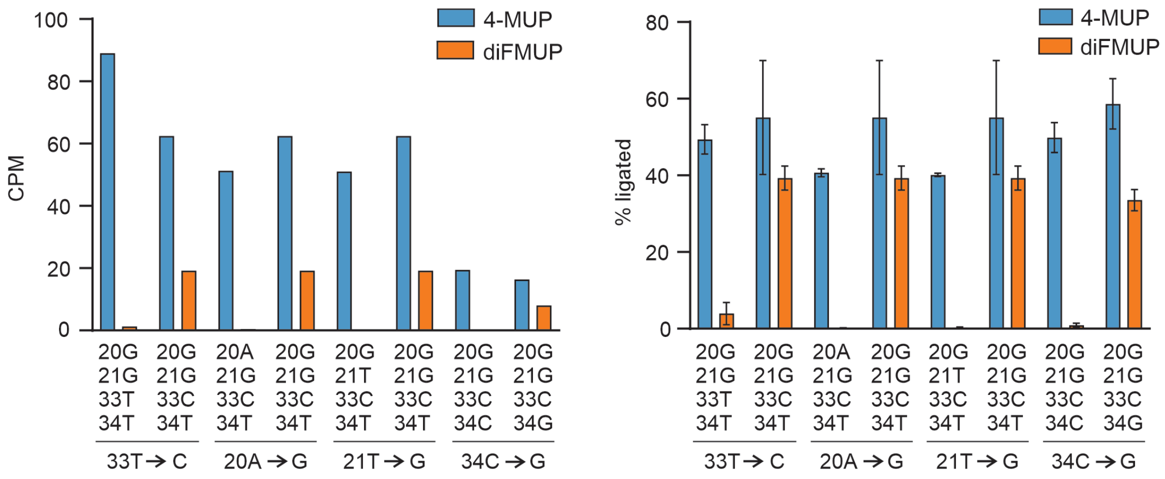

Figure S4. Mutant cycles showing the effects of mutations at positions 20, 21, 33 and 34 on the substrate specificity of Aurora. The activity of each mutant was determined by its CPM value after selection in the presence of 4-MUP or diFMUP (left graph) or using the ligation assay (right graph).

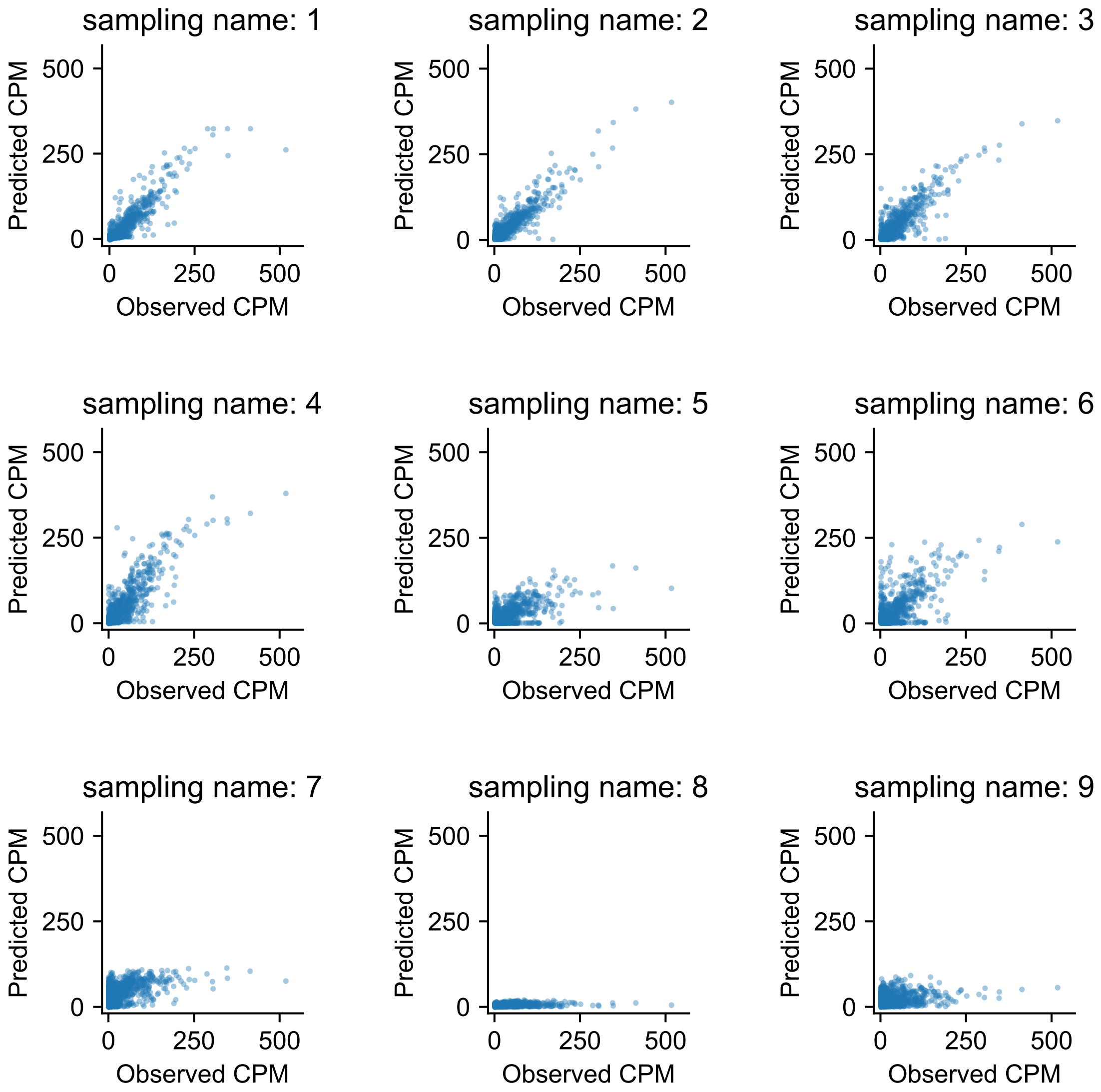

Figure S5. Predicted and experimentally observed CPM values calculated from the 4-MUP dataset using nine different training set sizes. See Table S3 for more information about training set sizes.

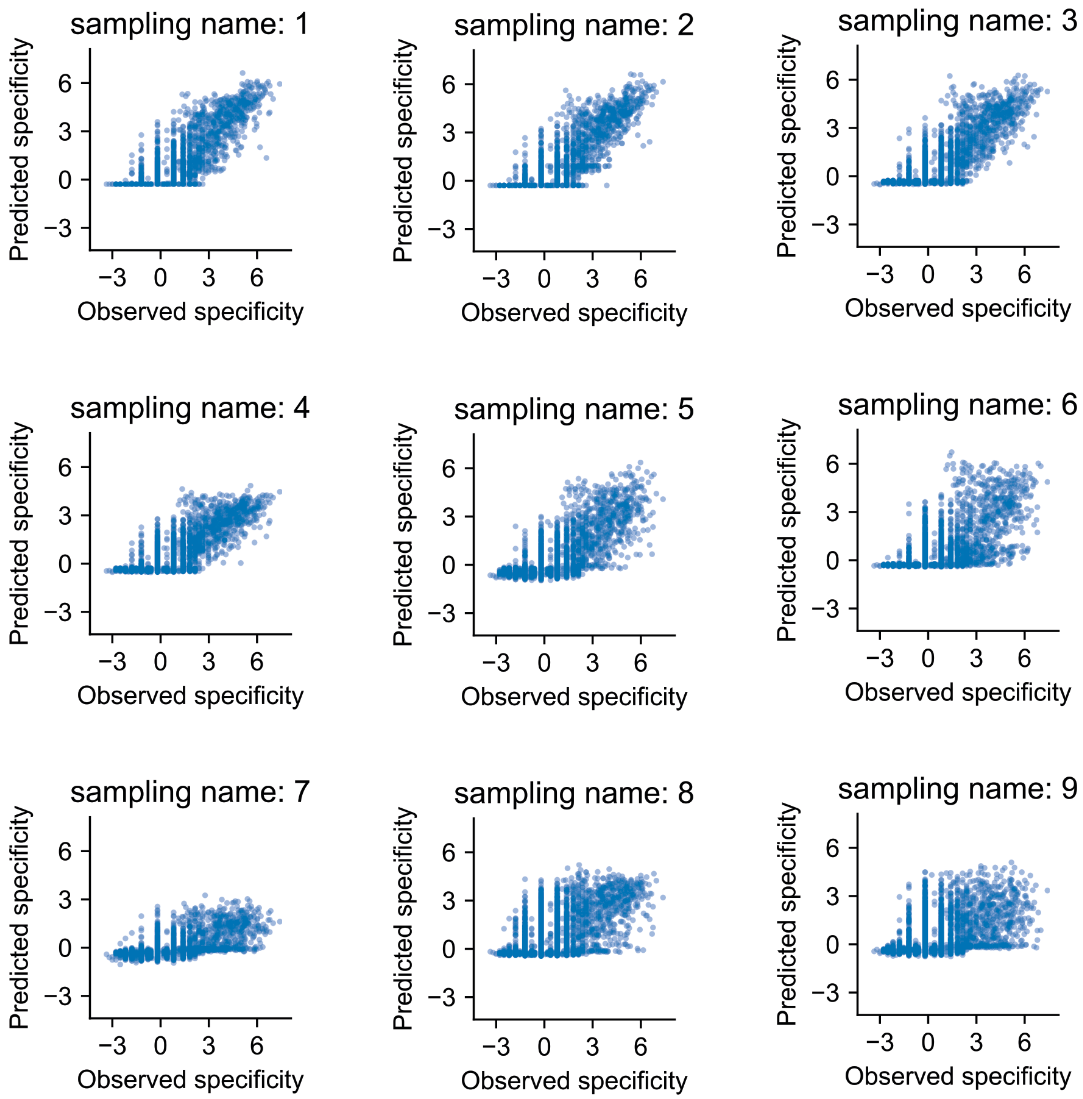

Figure S6. Predicted and experimentally observed specificity scores calculated from the 4-MUP and diFMUP datasets using nine different training set sizes. See Table S3 for more information about training set sizes.

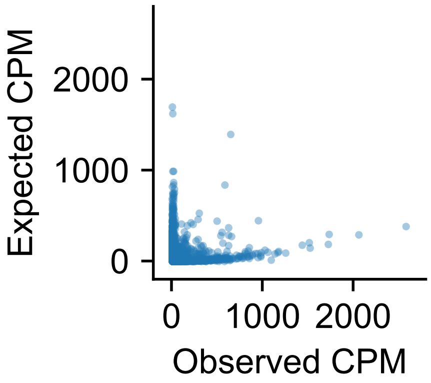

Figure S7. Calculation of predicted CPM values using an independence model. In this model, pairs of positions that form base pairs in Aurora are treated as units, but all other positions are treated as independent. To determine the expected CPM value of a sequence, the frequencies of mutations at variable positions (as determined by single-step selection in the presence of 4-MUP followed by high-throughput sequencing) are multiplied, and then converted into a CPM value. This model performed significantly worse than models constructed by machine learning.
